# Future Location Prediction for Emergency Vehicles Using Big Data: A Case Study of Healthcare Engineering

**DOI:** 10.1155/2020/6641571

**Published:** 2020-11-27

**Authors:** Muhammad Daud Kamal, Ali Tahir, Muhammad Babar Kamal, M. Asif Naeem

**Affiliations:** ^1^Institute of Geographical Information Systems, National University of Sciences and Technology, Islamabad, Pakistan; ^2^Department of Computer Science, COMSATS University, Islamabad, Pakistan; ^3^Department of Computer Science, National University of Computer and Emerging Sciences (NUCES), Islamabad, Pakistan; ^4^School of Engineering,Computer & Mathematical Sciences, Auckland University of Technology, Auckland, New Zealand

## Abstract

The number of devices equipped with GPS sensors has increased enormously, which generates a massive amount of data. To analyse this huge data for various applications is still challenging. One such application is to predict the future location of an ambulance in the healthcare system based on its previous locations. For example, many smart city applications rely on user movement and location prediction like SnapTrends and Geofeedia. There are many models and algorithms which help predict the future location with high probabilities. However, in terms of efficiency and accuracy, the existing algorithms are still improving. In this study, a novel algorithm, NextSTMove, is proposed according to the available dataset which results in lower latency and higher probability. Apache Spark, a big data platform, was used for reducing the processing time and efficiently managing computing resources. The algorithm achieved 75% to 85% accuracy and in some cases 100% accuracy, where the users do not change their daily routine frequently. After comparing the prediction results of our algorithm, it was experimentally found that it predicts processes up to 300% faster than traditional algorithms. NextSTMove is therefore compared with and without Apache Spark and can help in finding useful knowledge for healthcare medical information systems and other data analytics related solutions especially healthcare engineering.

## 1. Introduction

Analysing the movement pattern has always been of a keen area of interest, may it be automobile, humans, or any other moving object. These movement patterns can help analysts in making a decision related to the behaviour patterns of an object. For example, the idea of geo-marketing can be evolved if the pattern of the people who are shopping is observed.

Similarly, different location-aware applications can help urban planning by observing traffic patterns. Approximately 3.5 billion mobile phone users are predicted worldwide in 2020 [1]. A mobile user location is better estimated these days by the techniques that are currently being developed and used by the telecommunication providers. Therefore, mobile user's patterns and activities are sensed by using different mobility data records that are saved by telecommunication companies [2].

The objective of observing mobility data is to see why and when the objects move. To accomplish these objectives, various data sources are used such as Global System for Mobile communications (GSM) or Global Positioning System (GPS) for analysing and later transforming them into meaningful predicting patterns. The process of predicting patterns is known as Knowledge Discovery (KD), i.e., produced from raw data and converted into meaningful knowledge [3]. A hybrid system for location recognition and prediction which addressed key issues of location-based services, such as location recognition and prediction, was proposed by [4]. The system used a hybrid method combining *k*-Nearest Neighbour (kNN) and decision tree to effectively recognize the locations not only in the outdoor environment but also in the indoor environment. NextPlace was presented by [5], an approach for spatio-temporal user location prediction based on nonlinear analysis of the time series of start times and duration times of visits to significant locations. This approach allows forecasting not only the next location of a user but also his/her arrival and residence time, i.e., the interval of time spent in that location. With a particular objective to settle on an informed decision concerning which advancements to understand, information was assembled from a few previous literature studies.

The main motivation of this research is the availability of massive data with the industry which was never utilized for business intelligence in the context of Pakistan. The data has been gathered over the years and only used for real-time monitoring of vehicles. There is a huge potential to explore and perform geo visual analytics on available data on big data platforms such as Apache Spark.

The main purpose of this research is to analyse the spatio-temporal mobility patterns of Global Positioning System (GPS) data using new technologies for big data; i.e., Apache Spark is used to reduce the time taken per job for discovering useful information, which can help assist decision-making for real-world scenarios. The future location of vehicles is predicted from a large pool of data with more than 100 million rows of records after developing a novel algorithm, Next Spatio-Temporal Move (NextSTMove), on Apache Spark to optimize the time taken and later the predicted locations are verified against the real data.

The main contribution of our research is our newly proposed NextSTMove algorithm which is more efficient and accurate than existing algorithms. Moreover, we have used the real data of a local tracker company. The results of our algorithm can be very useful for long-term strategic and business advantages in healthcare engineering.

The remainder of this paper is structured as follows.

Section 2 describes the related literature review.  Section 3 presents a detailed methodology and proposed algorithm. Results and discussion are presented in Section 4. Finally, conclusions are outlined in Section 5.

## 2. Related Work

There are many trajectory prediction algorithms that exist in the literature. Over the years, various researchers have proposed novel algorithms which cater to their needs. Broadly, trajectory prediction algorithms are derived from machine learning approaches such as Bayesian networks [6], hidden Markov models [7], decision trees [8], neural networks [9], and state predictor methods [10]. This section describes existing work in this field while commenting on the above-mentioned parameters.

Research in mobility data is not that new. However, in the last few years, it has gained popularity for data mining and artificial intelligence, and health engineering [11–13]. Substantial amounts of information are produced by GPS and telecommunication technologies advancement. In the survey paper [14], five algorithms are used for four users that had different patterns. Innovations and advancement are giving hints of producing pervasive computing for mobility data which helps predict the accuracy. The trajectories that are stored for the semantics of mobility data are aiding in finding useful information about the movements of the objects [15].

Likewise, paper [16] presents visual techniques to generate trajectories (spatio-temporal sequences) using GPS data to assist in efficient trajectory projection of emergency vehicles in highly urbanized cities. Furthermore, papers [17, 18] use visual analysis to implement intelligent transportation enabling efficient utilization of new knowledge and complex data.

A spatial-temporal prediction method was proposed by [19] which is called Spatial-Temporal Recurrent Neural Networks (STRNN). The experimental results on real datasets showed that STRNN outperformed the state-of-the-art methods and can well model the spatial and temporal contexts. In [20, 21], authors discussed that with the growing data volume arises a need for processing spatio-temporal queries efficiently. For this, they used parallel processing in Secondo for geospatial big data analysis, while in [22] the context of time and space in a massive geospatial big data database is analysed using High-Performance Computing (HPC). A classification was presented by [23] for approaching decision trees to predict the next place of mobile users. The authors implemented an optimizer to find the best parameter combination for each user since users had widely varying behaviour. Finally, the performance of the approach was demonstrated by the results of the experiments on the real-life dataset of 80 mobile users provided by Nokia. The existing solutions for geolocation prediction (GP) and divided geolocation prediction into two primary parts were reviewed by [24]. The initial step proposed to manufacture a geolocation expectation show is Mining Popular Geolocation Region (MPGR), and the second is Mining Personal Trajectory (MPT). The results described the basic concepts of GP, the characteristics of MPGR, and MPT. They also discussed the limitations, openings, and future geolocation prediction analytical trends for mobility big data. Similarly, paper [25] proposed a methodology for the prediction of a user's outdoor location derived from contextual data (current location, day of the week, time, and speed), which were collected with a GPS device and with a smartphone. This methodology was based on spatial clustering of data and on-time segmentation to find points of interest that the user visits every day and every hour.

An investigation in 2013 by [26] worked on the perspectives identified with data accumulation and taking care of trajectories that are feeding to the databases with proper data. The trajectories recreation for producing meaningful trajectories includes procedures for gathering movement data and cleansing the data gathered, compression of data, and map coordinates to deliver noise-free trajectories. For the production of semantically compliant trajectories, raw spatial data from the common repository need to be recovered using different remaking tasks along with semantic trajectories. Moreover, paper [27] defined the concept behind the management of trajectory and their representations. The focus of the research was analysis on an extensive scale for phenomena related to mobility with more focus on the semantic behaviour of the data. The main goal of analysing the behaviour is indicating which behaviour defines which moving object.

An unprecedented amount of geospatial data gathered from moving objects defies human capability to analyse it. A study by [28] found new methods for processing and mining moving objects. For modelling and representing trajectories, paper [29] discusses the problem in the context of database systems. Moving objects databases represent a set of moving objects using abstract data types and maintain complete histories of movement.

An open-source software, Secondo, has a framework for big trajectory data whose data model is not fixed. This Database Management Systems (DBMS) prototype can be used for different data models. “WhereNext” are previously visited trajectory patterns that were extracted [30] that use previously extracted trajectory patterns. In most of the studies, few aspects of trajectory prediction are discussed. For example, some studies focus on indoor and outdoor navigation. Similarly, other studies highlight public and private datasets. In many studies, the authors have validated the accuracy of these algorithms on given datasets. In our research, we proposed a novel algorithm, NextSTMove, using Apache Spark to minimize the query and processing time for GPS big data. The reason is because Apache Spark is becoming de facto for processing big data in the computing world. We used it to predict future locations of vehicle GPS data.

Bayes-based predictors were used to add to the performance of their prediction for leveraging big data [31]. They studied a large Call Detail Record (CDR) dataset. At first, they explored the dataset and found that they can use call activity to generate prior probabilities for use in a Bayes predictor. With this reasoning, they developed an enhanced Bayes predictor that uses a distance threshold and the users' regular location to improve the generation of prior probabilities. Experimental results show that the enhancements they proposed increase accuracy of the Bayes based predictor by 17 percentage points. In the end, they concluded that it is feasible to leverage big cellular data to enhance location predictors without relying on external data.

Apache Spark developed in the year 2009 at Berley's lab [32] is said to have achieved the lowest latency rate in comparison with Secondo and Parallel Secondo. It is freely available for several operating systems such as Windows, Linux, and Mac Operating systems. Apache Spark is a Unified Analytics Engine for big data processing and management that supports streaming data, batched data, SQL, Graph, and machine learning processes. Apache Spark for point cloud spatial data management was used to achieve a lower latency rate [33]. They found, in comparison to the traditional methods for point cloud management, a file system storage, a single processing server, and a distributed approach based on Apache Spark were able to achieve a more agile speed and higher robustness and fault tolerance support. A comparison was made between the processing time taken by Relational Database Management System (PostgreSQL) and the time taken using Apache Spark. They achieved up to 300% of reduced latency rate, which shows Apache Spark is faster compared to these DBMS. As the number of nodes in the cluster was increased, the processing capabilities of the system increased. Increasing the number of points did not affect the query execution time of Apache Spark much, whereas queries run over PostgreSQL slow almost immediately. A new platform for geospatial big data was developed by [34] inside Apache Spark a GeoSpark SQL framework that was able to carry out geospatial SQL queries over an Apache Spark system.

The research showed that Apache Spark has a better performance than traditional Relational Database Management Systems (RDBMS) for a huge number of geospatial type queries. The methods for inserting the point data into the Apache Spark data structure are represented in [33]. The data were sliced into rectangular areas and each area was ingested in a separate document. Rectangular areas were numbered by a Geohash system and were stored in MongoDB. These structures allowed executions of operations by MapReduce for point cloud data, sometimes MongoDB or from an external framework like Apache Hadoop [35]. Similarly, paper [36] compared quadtree and R-tree on Spark for finding the difference in the query efficiency.

The purpose of this research is to analyse the spatial-temporal mobility patterns of GPS data using Apache Spark to reduce the time taken per job for discovering useful information, which can support the decision-making process for real-world problems.

## 3. Materials and Methods

### 3.1. Overview

In general, Apache Spark software is used for clustering of systems for very fast query response. It provides an executable environment for all the Spark applications in the Kernel of Spark core. The actual advantage of Apache Spark is that, compared with other technologies like Hadoop and MapReduce which only use disk for memory, Apache Spark uses memories and can also make use of the disk for the processes. Apache Spark is versatile, unlike the Hadoop ecosystem, as it does not have its own distributed file system but can make use of Hadoop Distributed File System (HDFS).

Apache Spark is a standalone software that does not use any resource manager. However, if we use it for more than one node and environment setup, we can use Yet Another Resource Negotiator (YARN) or Multiple Equivalent Simultaneous Offers (MESOs) for resource management, along with a distributed file system such as HDFS or Amazon Simple Storage Service (S3).

### 3.2. Spark SQL

Spark has a built-in library for processing structured data. This can be used for complicated SQL database queries and algorithm-based analytics. Spark SQL supports HIVE, SQL-like HiveQL query, Java Database Connectivity (JDBC), and Open Database Connectivity (ODBC). This can also enable some degree of connections with existing databases, warehouses, and business intelligence environments.

### 3.3. Deployment of Apache Spark

NextSTMove for predicting the future location of vehicles using Python programming is designed and implemented. PySpark utility was installed for Windows 7 using PIP. PySpark was locally installed in the system.

### 3.4. Approach

**Figure 1:**
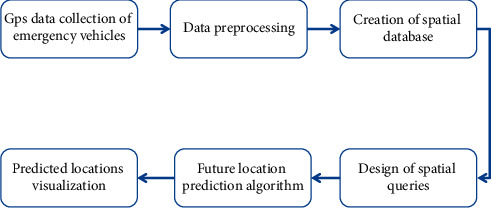
Detailed flowchart of methodology.

In this section, we describe the approach used for this research. The approach is divided into data collection, data pre-processing, creation of the spatio-temporal database in Apache Spark, creation of locations from user's data, and spatio-temporal queries. A flowchart of the methodology carried out is shown in Figure 1.

### 3.5. Raw GPS Data Collection

Real-time data from a vehicle GPS tracker company is used for this research.

The GPS data was received in MS-SQL database. The data spanned from 1st January 2016 and ended on 31st December 2016 with a total of 105,096,953 records in all twelve tables for each month of the year 2016. A total of 2261 vehicles contributed to the data.  Table 1 shows the data of the anonymized vehicle.

### 3.6. Data Pre-Processing

Data pre-processing involved cleaning of data, removal of unwanted data fields, and removal of missing fields to avoid null data in columns and adding new columns. Unlike [29] where the authors used an algorithm kNN to identify the latitudes and longitudes that are associated with each other, the data received were already assigned location names to a cluster of latitudes and longitudes which were quite accurate.

The GPS data received had thirty-three columns and most of them were not useful for the algorithm. To extract only the useful five columns from all the twelve tables of each month from the MS-SQL database to a single Comma Separated Values (CSV) file, the following batch command query was used on windows command-line environment:

*bcp* “*SELECT ReportGroupDate, vehicleRegistractionNo, locationName, latitude, longitude FROM GPSDataMonth1*

*union* “*SELECT ReportGroupDate, vehicleRegistractionNo, locationName, latitude, longitude FROM GPSDataMonth2*

*union* “*SELECT ReportGroupDate, vehicleRegistractionNo, locationName, latitude, longitude FROM GPSDataMonth3*

…

*union* “*SELECT ReportGroupDate, vehicleRegistractionNo, locationName, latitude, longitude FROM GPSDataMonth12*

queryout E:/TwelveMonthsTablesData.csv -t, -c -S . -d ReceievedGPSDatabase –-T

The data from each table of each month is now stored in one file and the total size of the data is reduced from 30 GB to 10 GB. There are more than 100 million records used in the Apache Spark database. Data within the CSV is arranged in the following sequence:

Date, Time, Day, vehicleRegistractionNo, LocationName, Latitude, Longitude

### 3.7. Creation of Spatial Database in Spark

A new database was created and then the data was stored in the Spark SQL table. PySpark syntax was used for storing the data in the Spark database. We used Spark Context to define the cores that are to be used by the local system. The Python command we used for this purpose is as follows:

*sc* *=* *SparkContext (*“*local[^∗^]*”,“*User*”)

*spark* *=* *SparkSession.builder*

*.master(*“*local*”)

*.appName (*“*Data cleaning*”)

.getOrCreate ()

We used DataFrames in Apache Spark version 2.0 Application Programming Interface (API) for managing our data. The final CSV file was generated for all the twelve months of the year and has been pre-processed and was assigned to a DataFrame using the following lines of code:

*SparkDataFrame* *=* *spark.read.format (*“*csv*”*).option (*“*header*”,“*true*”*).option (*“*mode*”,“*DROPMALFORMED*”*).load (*“*E:TwelveMonthsTablesData.csv*”)

*SparkDataFrame.createOrReplaceTempView (*“*TwelveMonthGPSData*”)

Here, SparkDataFrame is the DataFrame we are using and Spark's API to read the CSV file after loading it. A spatio-temporal database was created by using the createOrReplaceTempView library of Apache Spark. The ‘SparkDataFrame' was stored in our Spark SQL table which is used for NextSTMOve Algorithm.

### 3.8. Design of Spatial Queries

As the focus of this work is future location prediction using Apache Spark, the design of the query included the spatio-temporal aspect of the data, i.e., where and when. We queried for where a used vehicle will be at a given time and on a given day, for example, the location of a particular user, for example, “User A” on “‘Monday” between “9 Am” to “9 : 30 Am.”

We asked the user to input a valid vehicle number, the day they want to inquire, and the time between which they want to predict the vehicle location. After the user has input these parameters, a query gives the necessary results. The Spark SQL query is as follows:

*SparkDataFrame* *=* *spark.sql (*“*SELECT ^∗^ from TwelveMonthGPSData where vehicleRegistrationNo* *=* *'\%s' AND Day* *=* *'\%s' AND (Time* *=* *'\%s' or Time* *=* *'\%s')*” *\% (vehicleRegistrationNo, Day, TimeWindow1, TimeWindow2))*

### 3.9. Future Location Prediction Algorithm

As the DataFrame is updated and records all locations for the requested query. Each time a new location comes, it is considered as a key and their repetition is considered as tokens. The keys are checked against the total locations present in the DataFrame. Every time a key is repeated, respective tokens are also incremented. At the end of this nested loop, the count of each key is stored in their respective tokens. The top three keys having the most tokens are considered as the top three probable locations. These top three locations are stored for further processing.

Algorithm 1 explains the steps:

After the successful iterations of the above algorithm, three locations and the probability of their occurrences are output in the algorithm.

### 3.10. Creation of Location from Users Data

The results from the algorithm were visualized on the web using geospatial visualization libraries of Python. The result was extracted for web maps on run time to avoid any delays in data generation. The findings are discussed in Section 4.  Algorithm 2 explains the logical steps involved:

The coordinates of the top three locations along with their names and probabilities are ready to be mapped. Folium library in Python is used to generate a web map.

## 4. Results and Discussion

**Figure 2:**
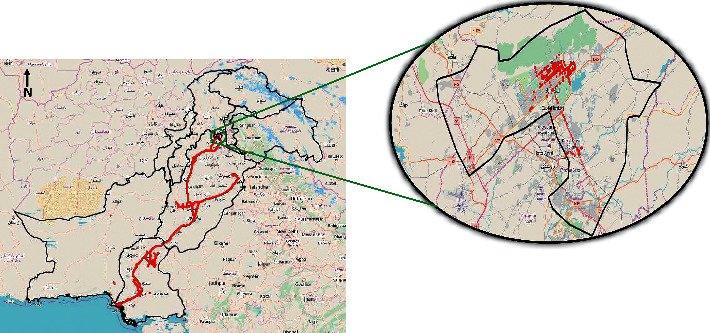
One month (January) data across Pakistan.

Figure 2 illustrates all the vehicles visited in one month. The visualization seems cluttered. Therefore, a further zoomed-in view on the city of a single user for the 1st month can also be seen.

### 4.1. Top Predicted Locations

**Figure 3:**
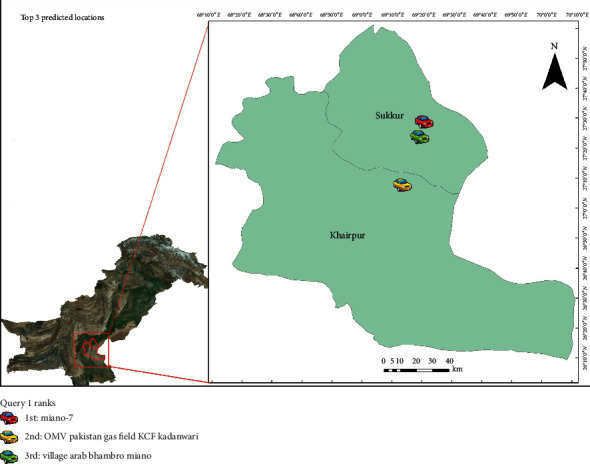
Results of query 1.

**Figure 4:**
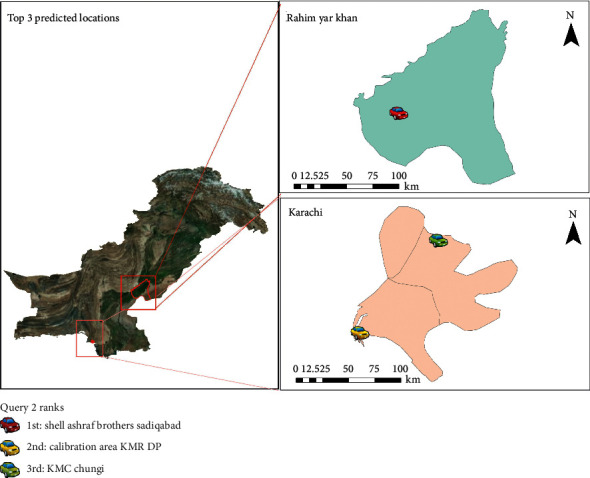
Results of query 2.

**Figure 5:**
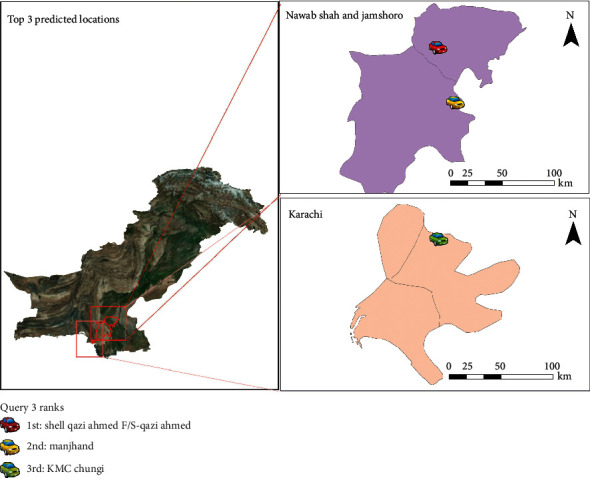
Results of query 3.

The queries were applied to the data to generate the top three probable locations between two-time intervals.  Table 2 shows the queries while Figures 3–5 display their results. The predictions of queries 2 and 3 are the same; however, the location predictions are quite apart because user B may be a frequent visitor of Karachi and interior province to provide ambulance services to the larger city from underdeveloped areas.

### 4.2. Latency for Apache Spark

A comparison is carried out on algorithms with and without Apache Spark. Initially, the algorithm was designed using a simple Python library. The NextSTMove algorithm was developed using queries and the latency of the process was calculated. The algorithm was then developed on Apache Spark using PySpark and queries applied are shown in Table 3. Up to 200 queries were applied and a sample of five random queries is shown in Table 3.

**Figure 6:**
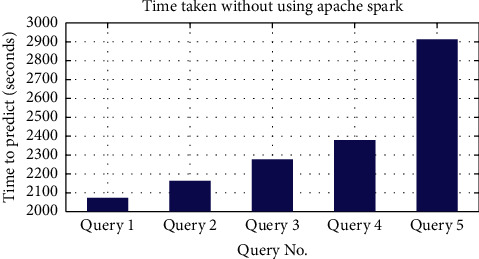
Time taken without using Apache Spark.

**Figure 7:**
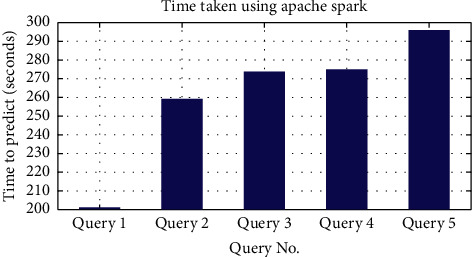
Time taken using Apache Spark.

After this, the algorithm was developed using Apache Spark for the queries shown in Table 3. We achieved a remarkable amount of decrease in the time taken by the queries. The job took more than two thousand seconds without using Apache Spark as illustrated in Figure 6. After using Apache Spark, the queries took less than 300 seconds.  Figure 7 illustrates the time taken in detail.

### 4.3. Accuracy of Predicted Locations

**Figure 8:**
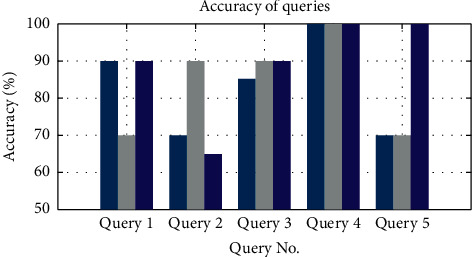
Accuracy of queries. Three predicted locations are shown for each query where the first, second, and third bars represent the first, second, and third locations, respectively.

Six months were used to predict the future locations of users and then the data from the next six months were used to find the accuracy of the predicted locations. We compared the real-time locations from the next six months' data with the predicted output for the queries. Table 3 shows the queries whose accuracy percentage is illustrated in Figure 8.

The three bars for each query in Figure 8 show the accuracy of the top three locations that were queried. As shown in Figure 8, query 4 has achieved a 100% accuracy, the reason being that this user has not changed his pattern for that time of the day. Therefore, the algorithm predicted it accurately. Similarly, in query 3 the algorithm achieves an accuracy of 90% for the top 2nd and 3rd predicted location, which depicts that this user was mostly using the same route, or he/she was present at the same location mostly.

The top 1st location for query 3 has a prediction rate of 85% which means the user showed varied behaviour.

## 5. Conclusions

Our work presents a novel algorithm, NextSTMove, where vehicle future movements are successfully predicted with and without Apache Spark. The algorithm achieved 75% to 85% accuracy and in typical cases 100% accuracy, where the users follow a repetitive pattern. The main aim of this research was to improve the latency and efficiency as compared to existing algorithms such as NextPlace [5]. Apache Spark, a big data platform, was fully utilized to achieve this. The algorithm reduced the processing time to up to 300%. This processing was done on a total of 2261 users having approximately 100 million data points.

This study is significant in predicting future locations of emergency vehicles. This can facilitate users to perform spatial tasks while improving the analytical knowledge gained from understanding their behaviours. The emergency vehicle tracker data reveal their spatio-temporal patterns. This research work can also help in solving many geospatial big data applications from both a commercial and security viewpoint.

As part of a future road map, we plan to expand our work by including real-time streaming of big data instead of processing with batched data only. Furthermore, we plan to introduce more nodes to the distributed processing to enhance the efficiency of the system running the queries. More data attributes can be introduced to analyse additional information which can reveal meaningful information and patterns for real-time applications.

Further analysis can be carried out in answering the question of how and why a user visited a particular location. This can help find the semantics of trajectories and carry out their analysis. Similarly, another future area in the algorithm can be predicting the next location of a vehicle using its previous history, i.e., where a user will be next after a specific location, by making a system to predict a route for vehicles that will be congested for a specific time and ask the emergency vehicles if they want to avoid that road. This study opens up further avenues for research. The main concern for using Apache Spark for NextSTMove is that during the loading of queries the first query takes more time to process as compared to the rest of the queries. Also, Apache Spark gets batched data, while other platforms such as Apache Flink can work with streaming data as well. Therefore, to increase processing capabilities, streaming data processing can be embedded along with it as part of our future work.

## Figures and Tables

**Algorithm 1:**
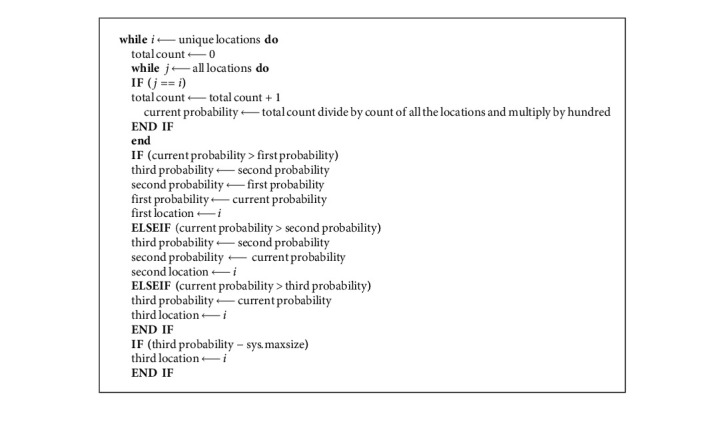
NextSTMove: algorithm for predicting the top three locations.

**Algorithm 2:**
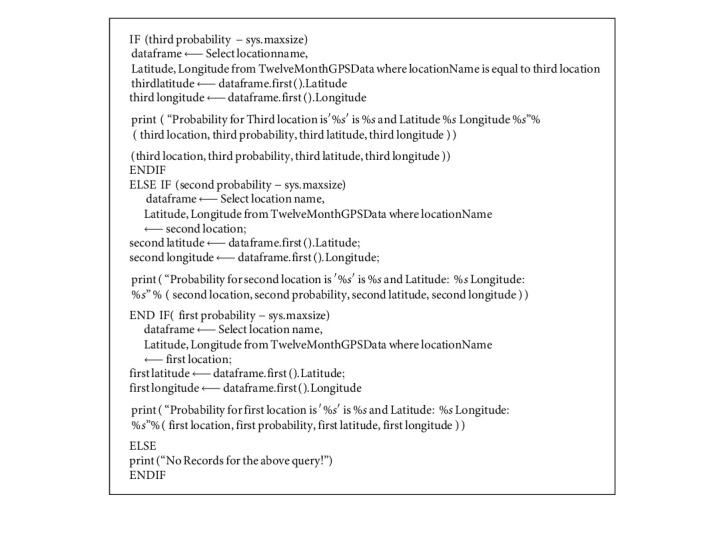
NextSTMove: location extraction from user's data.

**Table 1:** Raw data of a vehicle.

Veh. no.	Place of visit	Latitude	Longitude	Date	Day	Time
A	Subway Blue Area	33.71146667	73.0577	6/1/2016	Wednesday	00:00
A	Subway Blue Area	33.71146667	73.0577	6/1/2016	Wednesday	00:14
A	Jinnah super franchise	33.70936667	73.05383333	6/1/2016	Wednesday	00:21
A	Pak printing press	33.68015	73.07563333	6/1/2016	Wednesday	00:26
A	Shell quick fill F/Station	33.64775	73.0999	6/1/2016	Wednesday	00:31
A	Total Parco petrol diesel gas station	33.61301667	73.12583333	6/1/2016	Wednesday	00:36
A	TCS office	33.60621667	7 3.11566667	6/1/2016	Wednesday	00:41
A	Islamabad/Pindi airport	33.60576667	73.09916667	6/1/2016	Wednesday	00:46
A	Islamabad/Pindi airport	33.60641667	73.09966667	6/1/2016	Wednesday	00:51
A	Islamabad/Pindi airport	33.60628333	73.0989	6/1/2016	Wednesday	00:55

**Table 2:** Queries for predicting the top three locations.

Query no.	Vehicle no.	Day	Time from	Time to
Query 1	User A	Wednesday	12:00:00	01:00:00
Query 2	User B	Monday	09:00:00	10:00:00
Query 3	User B	Tuesday	09:00:00	10:00:00

**Table 3:** Queries with and without using Apache Spark.

Query no.	Vehicle no.	Day	Time from	Time to
Query 1	User A	Monday	10 : 00:00	12:00:00
Query 2	User B	Tuesday	11::00:00	13::00:00
Query 3	User B	Wednesday	12::00:00	14::00:00
Query 4	User D	Thursday	13::00:00	15::00:00
Query 5	User E	Friday	14::00:00	16::00:00

## Data Availability

Some sample data of a few vehicles might be provided on request.

## References

[1] News, I.67+ Revealing Smartphone Statistics for 2020, 2020

[2] Giannotti F., Pedreschi D. (2008). Mobility, data mining and privacy: geographic knowledge discovery.

[3] Renso C., Spaccapietra S., Zimányi E. (2013). *Mobility Data*.

[4] Cho S.-B. (2016). Exploiting machine learning techniques for location recognition and prediction with smartphone logs. *Neurocomputing*.

[5] Scellato S., Musolesi M., Mascolo C., Latora V., Campbell A. T. Nextplace: a spatio-temporal prediction framework for pervasive systems.

[6] Jensen F. V. (1996). *An introduction to Bayesian Networks*.

[7] Rabiner L., Juang B. An introduction to hidden Markov models. *IEEE ASSP Magazine1986*.

[8] Quinlan J. R. (1986). Induction of decision trees. *Machine Learning*.

[9] Specht D. F. (1990). Probabilistic neural networks. *Neural Networks*.

[10] Garnier J., Osguthorpe D. J., Robson B. (1978). Analysis of the accuracy and implications of simple methods for predicting the secondary structure of globular proteins. *Journal of Molecular Biology*.

[11] Chyu M. C., Austin T., Calisir F. (2015). Healthcare engineering defined: a white paper. *Journal of Healthcare Engineering*.

[12] Sharma A., Kumar R. Service-level agreement—energy cooperative quickest ambulance routing for critical healthcare services. *Arabian Journal for Science and Engineering2019*.

[13] Usman M. A., Philip N. Y., Politis C. 5G enabled mobile healthcare for ambulances.

[14] Petzold J., Bagci F., Trumler W., Ungerer T. Next Location Prediction within a Smart Office Building. *Cognitive Science Research Paper-University of Sussex CSRP2005*.

[15] Vieira M. R., Bakalov P., Tsotras V. J. On-line discovery of flock patterns in spatio-temporal data.

[16] Maqsood U., Tahir A., Fatima K. (2020). Interpreting rescue vehicle patterns using geovisual analytics for spatiotemporal resource allocation. *Arabian Journal of Geosciences*.

[17] Andrienko G., Andrienko N., Chen W., Maciejewski R., Zhao Y. (2017). Visual analytics of mobility and transportation: state of the art and further research directions. *IEEE Transactions on Intelligent Transportation Systems*.

[18] Andrienko N., Lammarsch T., Andrienko G. (2018). Viewing visual analytics as model building. *Computer Graphics Forum*.

[19] Liu Q., Wu S., Wang L., Tan T. Predicting the next location: a recurrent model with spatial and temporal contexts.

[20] Zhou C., Su F., Harvey F., Xu J. (2016). *Spatial Data Handling in Big Data Era*.

[21] Ashfaq M., Tahir A., Orakzai F. M., McArdle G., Bertolotto M. (2017). Using T-Drive and BerlinMod in parallel SECONDO for performance evaluation of geospatial big data processing. *In Spatial data handling in big data era*.

[22] Li Z. (2020). Geospatial Big Data Handling with High Performance Computing: Current Approaches and Future Directions. *In High Performance Computing For Geospatial Applications*.

[23] Tran L. H., Catasta M., McDowell L. K., Aberer K. Next place prediction using mobile data.

[24] Xu G., Gao S., Daneshmand M., Wang C., Liu Y. (2016). A survey for mobility big data analytics for geolocation prediction. *IEEE Wireless Communications*.

[25] Guessoum D., Miraoui M., Tadj C. (2016). Contextual location prediction using spatio-temporal clustering. *International Journal of Pervasive Computing and Communications*.

[26] Marketos G., Damiani M. L., Pelekis N., Theodoridis Y., Yan Z. (2013). *Trajectory Collection and Reconstruction*.

[27] Spaccapietra S., Parent C., Damiani M. L., de Macedo J. A., Porto F., Vangenot C. (2008). A conceptual view on trajectories. *Data & Knowledge Engineering*.

[28] Morzy M. Prediction of moving object location based on frequent trajectories.

[29] Güting R. H., Behr T., Düntgen C. (2010). SECONDO: a platform for moving objects database research and for publishing and integrating research implementations. *IEEE Data Engineering Bulletin*.

[30] Monreale A., Pinelli F., Trasarti R., Giannotti F. Wherenext: a location predictor on trajectory pattern mining.

[31] Matekenya D., Ito M., Shibasaki R., Sezaki K. Enhancing location prediction with big data: evidence from dhaka.

[32] Spark A. (2019). Mobile subscriptions near the 7-billion mark. *Does Almost Everyone Have a Phone?*.

[33] Paji ´c V., Govedarica M., Amovi ´c M. (2018). Model of point cloud data management system in big data paradigm. *ISPRS International Journal of Geo-Information*.

[34] Huang Z., Chen Y., Wan L., Peng X. (2017). GeoSpark SQL: an effective framework enabling spatial queries on 345 spark. *ISPRS International Journal of Geo-Information*.

[35] Boehm J., Liu K., Alis C. Sideloading-ingestion of large point clouds into the apache spark big data engine.

[36] Xie X., Xiong Z., Hu X., Zhou G., Ni J. On massive spatial data retrieval based on spark.

